# Further evidence associating IgG1, but not IgG2, with susceptibility to canine visceral leishmaniasis caused by *Leishmania (L.) infantum chagasi*-infection

**DOI:** 10.1051/parasite/2017039

**Published:** 2017-10-13

**Authors:** Luciana Vieira do R. Lima, Liliane Almeida Carneiro, Marliane Batista Campos, Thiago Vasconcelos dos Santos, Patrícia Karla Ramos, Márcia Dalastra Laurenti, Claudio Eduardo C. Teixeira, Fernando T. Silveira

**Affiliations:** 1 Parasitology Department, Evandro Chagas Institute (Surveillance Secretary of Health, Ministry of Health), Ananindeua, Pará State Brazil; 2 National Center of Primates (Surveillance Secretary of Health, Ministry of Health), Ananindeua, Pará state Brazil; 3 Pathology Department, Medical School of São Paulo University São Paulo, São Paulo State Brazil; 4 Tropical Medicine Nucleus, Federal University of Pará, Belém, Pará State Brazil

**Keywords:** IgG1/IgG2 responses, *Leishmania (L.) infantum chagasi*, canine visceral leishmaniasis, Brazilian Amazon

## Abstract

We present here a cross-sectional study analyzing the IgG1 and IgG2 immune responses to natural canine *Leishmania (L.) infantum chagasi*-infection and their relationships with delayed-type hypersensitivity (DTH) in 50 mongrel dogs with previous positive serodiagnoses (IFAT-IgG) (56% with subclinical status [= apparently healthy] and 44% clinically sick), living in endemic areas for visceral leishmaniasis in the Brazilian Amazon. IgG1 and IgG2 responses were measured using commercial polyclonal antibodies in ELISA, while DTH was elicited by intradermal skin test using cultured promastigotes *L. (L.) i. chagasi*-antigen. Data analyses used Chi-square and Pearson's *r* coefficient (95% confidence interval). Regarding DTH and the clinical statuses of dogs, it was noted that 100% of the animals showing positive DTH (*n* = 8) were from the subclinical group, while 100% showing negative DTH were from the clinically sick group; higher IgG2 than IgG1 responses were observed in both clinical groups. However, when this comparison was made between the subclinical and sick groups, higher IgG1 responses were noted in the dogs from the sick rather than the subclinical group, while no differences were noted between the IgG2 responses in the dogs from both clinical groups. Additionally, we found lower IgG1 responses in dogs from the subclinical group showing positive DTH than in the dogs from the subclinical or sick groups with negative DTH; no differences were found between the IgG2 responses of these two clinical groups. These findings suggest that the IgG1, but not the IgG2, response is associated with susceptibility to canine visceral leishmaniasis (CVL).

## Abstract (fr)

Nous présentons ici une étude transversale analysant les réponses immunitaires IgG1 et IgG2 à l'infection canine naturelle par *Leishmania (L.) infantum chagasi* et leurs relations avec une hypersensibilité retardée (HR) chez 50 chiens errants avec des sérologies positives antérieures (IFAT-IgG) (56 % avec statut sous-clinique [= apparemment en bonne santé] et 44 % malades cliniquement) vivant dans une zone endémique de leishmaniose viscérale dans l'Amazonie brésilienne. Les réponses IgG1 et IgG2 ont été mesurées en utilisant des anticorps polyclonaux commerciaux en ELISA, tandis que l'HR a été provoquée par un test cutané intradermique utilisant un antigène de promastigotes cultivés de *L. (L.) i. chagasi*. Les analyses de données ont utilisé le coefficient de Chi-carré et Pearson (intervalle de confiance de 95 %). En ce qui concerne l'HR et les états cliniques des chiens, on a noté que 100 % (*n* = 8) des animaux présentant une HR positive provenaient du groupe sous-clinique, tandis que 100 % montrant une HR négative provenaient du groupe cliniquement malade. Des réponses IgG2 supérieures aux IgG1 ont été observées dans les deux groupes cliniques. Cependant, lorsque cette comparaison a été faite entre les groupes sous-clinique et malade, des réponses IgG1 plus élevées ont été observées chez les chiens du groupe malade que chez le groupe sous-clinique, alors qu'aucune différence n'a été notée entre les réponses IgG2 chez les chiens des deux groupes cliniques. En outre, nous avons trouvé des réponses IgG1 plus faibles chez les chiens du groupe sous-clinique présentant une HR positive que chez les chiens des groupes sous-cliniques ou malades avec HR négative. Aucune différence n'a été trouvée entre les réponses IgG2 de ces deux groupes cliniques. Ces résultats suggèrent fortement que la réponse IgG1, mais pas IgG2, est associée à une susceptibilité à la leishmaniose viscérale canine.

## Introduction

American canine visceral leishmaniasis (CVL) is a parasitic zoonosis widely distributed in Latin America and caused by *Leishmania (L.) infantum chagasi* (**= ***Leishmania chagasi* Cunha & Chagas 1937), a protozoan of the order Kinetoplastida, family Trypanosomatidae, and genus *Leishmania* [15,19,27]. CVL and human American visceral leishmaniasis (AVL – a disease caused by the same *Leishmania* species) are naturally transmitted by *L. (L.) i. chagasi*-infected phlebotomine sand fly – *Lutzomyia longipalpis* (Psychodidae: Phlebotomine) – the most important vector in Brazil [14]. Taking into account the epidemiology of these diseases, it seems reasonable to assume that CVL is more prominent than AVL due to its higher prevalence, as well as the fact that both subclinical (**= **apparently healthy) and clinically sick dogs may act as sources of infection of the phlebotomine vector [17,30]. Additionally, in light of the high prevalence of CVL in endemic AVL areas, the persistent skin parasitism found in the infected dogs, and the emergence of CVL earlier than AVL, there can be no doubt about the important role of dogs in the epidemiology of AVL. The estimated canine/human infection ratio was 4:1 in the Brazilian Amazon [29].

With regards to the clinical features of canine *L. (L.) i. chagasi*-infection, there is a consensus that infected dogs can show a spectrum of clinical signs varying from subclinical to severe terminal illnesses that usually culminate in death. These clinical signs include lymphadenopathy, periorbital and nasal dermatitis, onychogryphosis, fever, apathy, diarrhea, intestinal hemorrhaging, loss of weight, splenomegaly, and hepatomegaly. Ulceration of the nose, ears, and tail are also frequently observed [12]. Additionally, there is some evidence from canine *Leishmania (L.) infantum*-infection in the Mediterranean region of Europe showing that subclinical infected dogs can convert to diseased animals following periods ranging from three months to seven years [20,31]. In the Brazilian Amazon, a recent investigation that followed a dog cohort for two years reported a 35.6% rate of subclinical dogs that converted to diseased states [7].

In contrast with the murine model of *Leishmania (L.) major*, no consistent associations have yet been described concerning IgG1 and IgG2 subclass ratios and disease resistance in canine infection – suggesting that those inconsistent results may partially reflect a low specificity of *Leishmania*-antigenic preparations and/or commercial antibodies. As such, a previous study carried out in Pará State in the Brazilian Amazon that examined IgG1-IgG4 responses in a cohort of 57 naturally infected dogs (seropositive for IgG) demonstrated that all IgG subclasses, detected by a panel of monoclonal antibodies, were higher in sick dogs than in healthy animals, suggesting that CVL is associated with the upregulation of all IgG subclasses, particularly IgG1, IgG3 and IgG4 [25]. Additionally, a new study used stored sera from the same dogs of the previous study and IgG1 and IgG2 responses to crude *Leishmania*-antigens were specifically measured using commercial polyclonal antibodies, revealing that both IgG1 and IgG2 subclasses had higher titers in clinically sick dogs than in healthy dogs. This showed general upregulation of IgG in these infected animals [8]. A survey undertaken in São Paulo State (southeastern Brazil), however, identified the likely dominance of particular IgG subclasses in 120 sera from confirmed seropositive dogs as evaluated by ELISA, demonstrating a dominant IgG1 response in subclinical dogs. Diseased dogs showed a mixed pattern of IgG subclass productions [23].

Studies of CVL immune responses to canine *L. (L.) infantum*-infections in the Mediterranean region showed definite similarities to the results of Brazilian research concerning the IgG1 and IgG2 canine immune responses to *L. (L.) i. chagasi*-infection. One of these European reports analyzed IgG subclass responses using ELISA, and concluded that dogs produced both IgG1 and IgG2 antibodies, with IgG2 being associated with subclinical infections and IgG1 with disease expression, suggesting that the differential responses indicated dichotomous immune responses [10]. In another study carried out in Lyon, France, IgG1 and IgG2 levels were examined in 14 dogs (six subclinical and eight sick) using ELISA. The assays detected both IgG1- and IgG2-specific antibodies in fourteen infected dogs – but the IgG2 subclass appeared to be predominant [3]. In Barcelona, Spain, IgG1 and IgG2 expressions were examined in 109 sera from 50 dogs using ELISA and were then compared to clinical conditions (subclinical or sick). IgG1 and IgG2 responses of clinical and subclinical infections were found to differ mainly in terms of subclass production values, with higher IgG2 production occurring especially in sick dogs [13]. Additionally, IgG1 and IgG2 responses were measured and compared in Trás-os-Montes and Alto Douro, Portugal, with the delayed-type hypersensitivity (DTH) response in three groups of dogs clinically and serologically characterized as:

- subclinical and direct agglutination test (DAT)-seronegative;
- subclinical and DAT-seropositive;
- DAT-seropositive and sick. IgG2 was considered a marker of disease, as significantly higher levels of this subclass were recorded in sick dogs [6].

Thus, regarding the inconsistent associations concerning resistance or susceptibility markers to CVL based on IgG1 and IgG2 responses, we decided to analyze these IgG subclass responses taking into account their relationships with the clinical statuses and DTH responses of infected dogs. This was the focus of the present study designed to amplify our understanding of the clinical-immunological parameters of canine infection. This approach was designed to address specific questions concerning parasite interactions with canine immune responses with the aim of contributing to new control strategies against the disease.

## Materials and methods

### Ethics approval

This work was undertaken in accordance with standard ethics criteria for scientific research using animals. The project was approved by the Ethics Committee for Animal Use in Research of the Evandro Chagas Institute, license No. 007/2012. The dog owners were informed of the objectives of the study and consented to, and signed, a free consent and understanding agreement.

### Study area

Our study was undertaken in an endemic area for AVL in Vila Santana do Cafezal, located approximately 7 km from the administrative center of the municipality of Barcarena (01° 30' S** – **48° 37' W), Pará State, Brazil. This area was chosen because it demonstrates ecological and social economic conditions favorable to AVL transmission. These aspects were described in earlier studies that recorded 85% prevalence of the phlebotomine vector *Lu. longipalpis* in and around human habitations in this area [33], as well as a canine infection prevalence of 43% (based on humoral responses by the indirect fluorescence antibody test [IFAT-IgG] and cellular responses by DTH) [29].

### Study design and the canine population examined

The study was designed as a cross-sectional examination of a population of 50 dogs showing positive serodiagnoses for CVL (IFAT-IgG) that had been randomly selected during an epidemiological survey which examined 316 dogs for CVL (July/August 2012) in the village Santana do Cafezal, municipality of Barcarena, Pará State, Brazil [7]. In addition to the IFAT-IgG, leishmanin skin tests (LST) were performed on the dogs, and eight of 50 also showed LST reactivity (= DTH positive).

After the selection of 50 dogs showing immunological diagnoses of infection (being 100% seropositive [IFAT-IgG] and 16% DTH positive), their serum samples were also ELISA (IgG) tested, which confirmed their serological reactivity and their inclusion in the present study. The serum samples were subsequently submitted to ELISA testing against the polyclonal antibodies IgG1 and IgG2, following previous research [16,18,22], to better evaluate the responses of these subclasses to natural infections as well as to correlate the responses with the clinical states and cellular DTH responses of the dogs.

### Clinical evaluations and the characteristics of infected dogs

As described above, all of the 316 dogs included in the research design that had been diagnosed for natural infection were clinically evaluated by a veterinarian, who recorded the clinical signs of each animal individually. After clinical inspection, the animals were classified into two groups: a subclinical group (SCG) – infected, but without clinical signs of illness (= apparently health); and diseased cinical group (DCG) – infected, and showing clinical signs of illness (= sick) [32]. Among the 22 clinically ill dogs, the most frequent clinical signs encountered were: thinning, cachexia, dermatitis, onychogryphosis, skin ulcers, hepatomegaly, splenomegaly, and lymphadenopathy.

As such, the dog population in the present study (*n* = 50) comprised 28 SCG (56%) and 22 DCG animals (44%). Of the 28 SCG dogs, 20 (71.4%) were males and eight (28.6%) were females; of the 22 DCG animals, 10 (45.5%) were males and 12 (54.5%) were females. In terms of their ages, of the 28 SCG animals, 26 (92.8%) were < 7 years, and two (7.2%) were ≥ 7 years; among the 22 DCG dogs, 20 (90.9%) were < 7 years, and two (9.1%) were ≥ 7 years.

### Collection of tissue samples

Blood samples were taken from dogs in the study area with the consent of their owners, who also signed a free consent and understanding agreement. For the serological testing, the dogs were immobilized and a 3 mL blood sample was taken intravenously (cephalic vein). The samples were then stored, without anticoagulants, to obtain the serum samples; the material was subsequently kept at −20 °C until use.

### Immunological test procedures

In order to optimize the enzyme-linked immunosorbent assay (ELISA), we tested different soluble concentrations of promastigote-antigens of the same *L. (L.) i. chagasi* strain (MCAO/BR/2010/M27840/Barcarena, Pará) used for the LST and IFAT assays in previous epidemiologic surveys [7], as well as different dilutions of control positive sera with low, moderate, and high titers, alkaline phosphatase conjugates, and cut-off levels. After checkerboard titrations, the best discriminative conditions between positive and negative controls for each isotype were defined, and the ELISA performed as follows: a suspension of stationary-phase promastigote was disrupted by freeze-thawing, then centrifuged at 10 000 *g* for 30 min at 4 °C. To determine antigen concentrations, standardizations were performed by constructing a standard curve using different concentrations, which provided a final protein concentration of 400 μg/mL using the Bradford method [4]; the samples were then stored at −20 °C until use.

The microplates were coated with 10 μg/mL of the soluble IgG antigen in 0.1 M carbonate-bicarbonate buffer (pH 9.5), or with 20 μg/mL for IgG1 and IgG2, and held at 4 °C overnight. After blocking the wells with 10% bovine albumin solution PBS with added 0.05% Tween (PBS-T), 100 μL of the diluted serum samples (at ratios of 1:400 to 1:6400 for IgG, and 1:200 to 1:3200 for both IgG1 and IgG2) were added to each well (two-fold dilutions) and incubated at 37 °C for 2 hours. After washing with PBS-T, 100 μL of alkaline phosphatase-labeled secondary antibody solutions at dilutions of 1:2000 for IgG, and 1:500 for both IgG1 and IgG2 (Bethyl Laboratories, USA) in PBS-T were added to each well and the plates incubated at 37 °C for 1 h. After further washing, 100 μL/well of 1.0 mg/mL pNPP (Sigma, USA) in 0.1 M pH 9.5 carbonate-bicarbonate buffer was added, and the samples incubated for 30 min at room temperature. The reactions were halted with 50 μL/well of 1 M NaOH, and absorbance was subsequently measured at 405 nm using an ELISA reader. The minimum detection level (cut-off) was set at the mean optical density obtained from the negative controls plus three standard deviations [16,18,22].

The negative control (*n* = 5) and test (*n* = 50) serum samples were serially diluted (base 2), in duplicate. The results were expressed using the serological titration method for ELISA, that is, the inverse of the last dilution in which the sera demonstrated greater absorbance than the cut-off value, which were calculated for each of the negative sera control dilutions [9]. To interpret the results, the mean values of the absorbencies of the test samples (at each dilution level) were compared to the cut-off value until the final point could be determined (the first dilution in which the mean absorption was less than the cut-off point). The titer was considered the inverse of the last dilution before the final point. The cut-off of each solution was determined by the mean of the absorption of the negative serum controls plus three standard errors.

### Data analysis

Log_10_ transformations of the sera titer values obtained using ELISA were used in all analysis stages. We tested the null hypothesis that the differences of proportions observed between variables in the study were not significant using Fisher's exact test (*α* = 0.05). Thus, we evaluated possible discrepancies between proportions, which could either indicate selection bias or suggest associations between the variables. Additionally, two-way Analysis of Variance (ANOVA) (*α* = 0.05), followed by Turkey's multiple comparisons test (*α* = 0.05), were used to compare the mean values of the sera titer of the different groups; 95% confidence intervals of the differences between the mean sera titer values were also calculated. Furthermore, we evaluated possible correlations between the values of IgG, IgG1, and IgG2 using Pearson's linear correlation coefficient (*r*), a paired *t* test (*α* = 0.05) to evaluate the null hypothesis that *r* is not different from zero; the coefficient of determination (R^2^) was used to evaluate how variables share their variability, and the 95% confidence interval of *r*. All data analyses were performed using GraphPad Prism version 6.00 for Mac (GraphPad Software, La Jolla, California USA, www.graphpad.com).

## Results

### Sample validation

**Figure 1:**
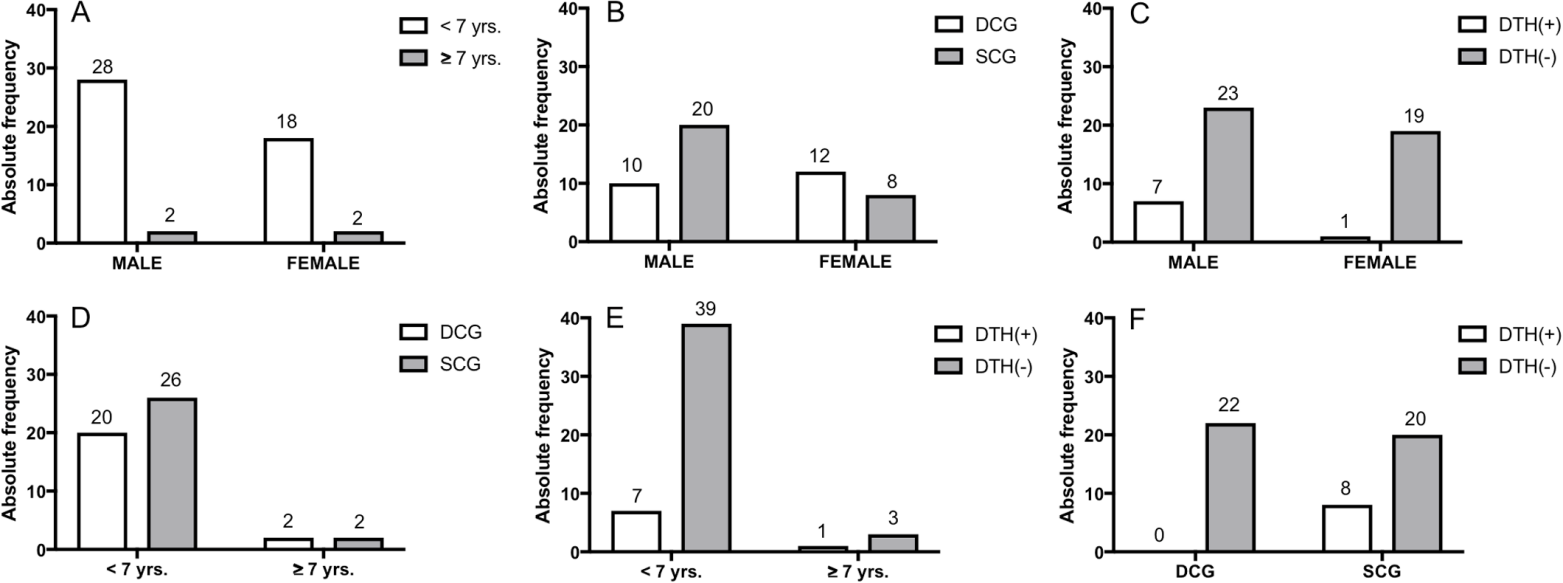
Above: frequencies of male and female dogs in relation to age (A), their clinical states (B), and their DTH results (C). Below: frequencies of the ages of the dogs in terms of their clinical states (D), and their DTH results (E), in addition to the frequency of sick dogs (DCG) and apparently healthy animals (SCG) as a function of their DTH results (F).

In general, no evidence of discrepancies between the observed frequencies in the dataset was found (Figure 1). In other words, no significant differences were found between frequencies of male and female dogs considering their age (Figure 1A, *p *> 0.9999), clinical signs (Figure 1B, *p* = 0.0845), and DTH results (Figure 1C, *p* = 0.1227). Likewise, no significant differences were found between frequencies of dogs considering jointly their age and clinical states (Figure 1D, *p *> 0.9999), and their age and DTH results (Figure 1E, *p* = 0.514). However, a statistical discrepancy between the frequencies of dogs considering jointly their clinical states and DTH results was found (Figure 1F, *p* = 0.0064). Instead of a selection bias, this statistical discrepancy reflected an epidemiological situation: in the original sample of 30 DCG-dogs from Vila Santana do Cafezal (Barcarena municipality, Pará State, Brazil, where the sampling was undertaken), no DTH**^(+)^** dogs were found. Therefore, this statistical discrepancy reinforces the notion that DTH**^(+)^** is associated with SCG-dogs.

### Evaluation of IgG, IgG1, and IgG2 responses by ELISA within the clinical groups of infected dogs

**Figure 2:**
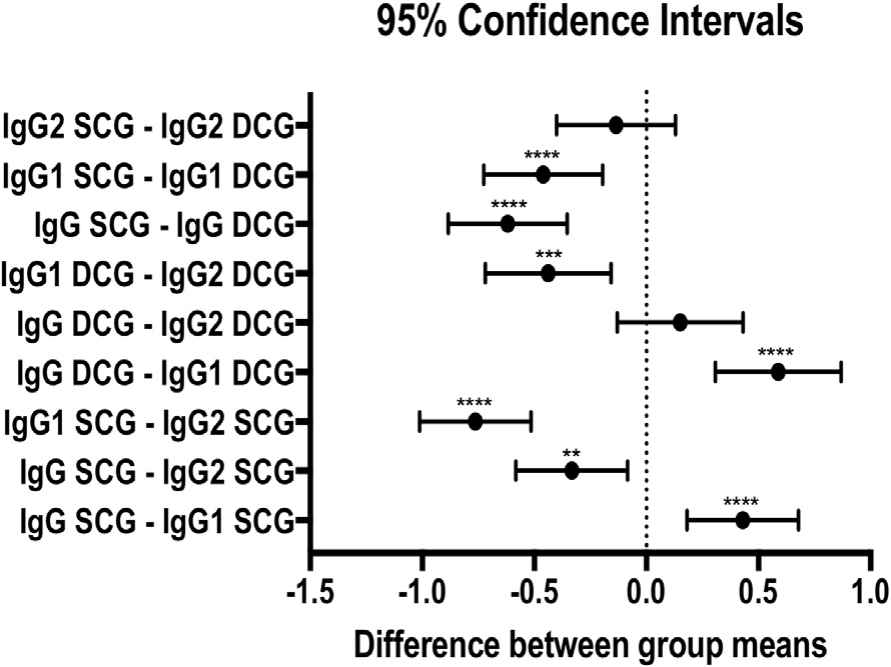
The 95% confidence intervals of the differences between the mean values of the IgG, IgG1, and IgG2 titrations obtained by ELISA, considering the variable of clinical state. The vertical dotted line indicates the absence of any differences between the means evaluated. ^**^, *p* = 0.0022; ^***^, *p* = 0.0002; ^****^, *p* < 0.0001.

Figure 2 shows log_10_ values of serological titers of IgG, IgG1, and IgG2 of sick animals (DCG) and subclinical animals (SCG). On average, intragroup comparisons demonstrated that IgG values were greater than IgG1 values in both groups (*p* < 0.0001), and IgG2 values were also greater than IgG1 values in both groups (SCG: *p* < 0.0001; DCG: *p* = 0.0002). Only the SCG group showed IgG2 values greater than IgG values (SCG: *p* = 0.0022; DCG: *p* = 0.7274). Additionally, it can be observed in Figure 2 that, on average, intergroup comparisons demonstrated that IgG and IgG1 values were greater in the DCG group (*p* < 0.0001). There were no statistically relevant differences between the IgG2 values in the groups evaluated (*p* = 0.7818).

**Figure 3:**
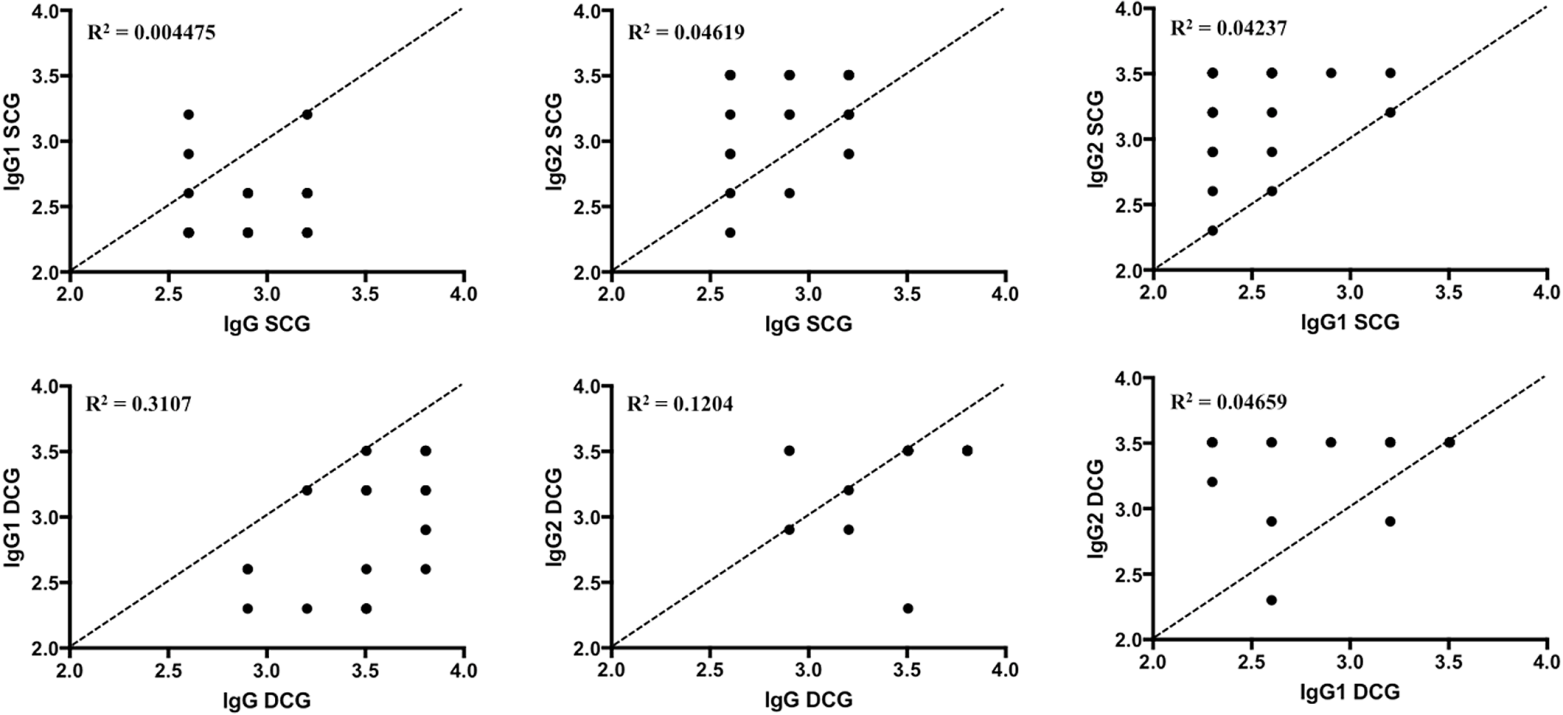
Correlation of log_10_ transformations of sera titer values of IgG, IgG1, and IgG2 of the SCG (top) and DCG (bottom) groups obtained using ELISA. Diagonal dashed lines indicate what would be a perfect positive correlation (*r* = 1) between the variables.

We also evaluated the degree of correlation between serological titers of IgG, IgG1, and IgG2 within the DCG and SCG groups, and no relevant correlation was found (Figure 3). However, the *p* value described in Table 1 suggests that, not by chance, there is a statistically positive correlation in the DCG group between the IgG and IgG1 values (*p* = 0.007). In addition, although the amplitude of the confidence interval of *r* coefficient (Table 1) indicates that this statistical correlation is not clinically significant, the determination coefficient (R^2^ = 0.3107) indicates that IgG and IgG1 within the DCG group are well correlated, e.g., vary together in a similar fashion, differently from IgG and IgG1 (R^2^ = 0.004475) and IgG and IgG2 (R^2^ = 0.04619) in the SCG group, and also differently from IgG and IgG2 values (R^2^ = 0.1204) in the DCG group. Finally, it is also worth noting (Figure 3) that IgG values were generally greater than IgG1 values within all groups, and that IgG2 values were generally larger than IgG and IgG1 values, corroborating the results presented in Figure 2.

**Table 1:** Analyses of correlations between IgG, IgG1, and IgG2 as a function of the variable of clinical state.

Statistics	Correlations	SCG	DCG
*r*	(IgG_,_ IgG1)	0.0669	0.5574
	(IgG_,_ IgG2)	0.2149	0.347
	(IgG1_,_ IgG2)	0.2058	0.2158

95% CI	(IgG_,_ IgG1)	−0.314 to 0.4293	0.1775 to 0.7927
	(IgG_,_ IgG2)	−0.1719 to 0.5443	−0.08744 to 0.6705
	(IgG1_,_ IgG2)	−0.1811 to 0.5376	−0.2264 to 0.5843

*p* value	(IgG_,_ IgG1)	0.7352	0.007(^*^)
	(IgG_,_ IgG2)	0.2721	0.1137
	(IgG1_,_ IgG2)	0.2933	0.3347

(^*^) Numerically positive linear correlation present, but not significant.

### Evaluations of IgG, IgG1, and IgG2 responses by ELISA, and their relationships with DTH expression within the clinical groups of infected dogs

**Figure 4:**
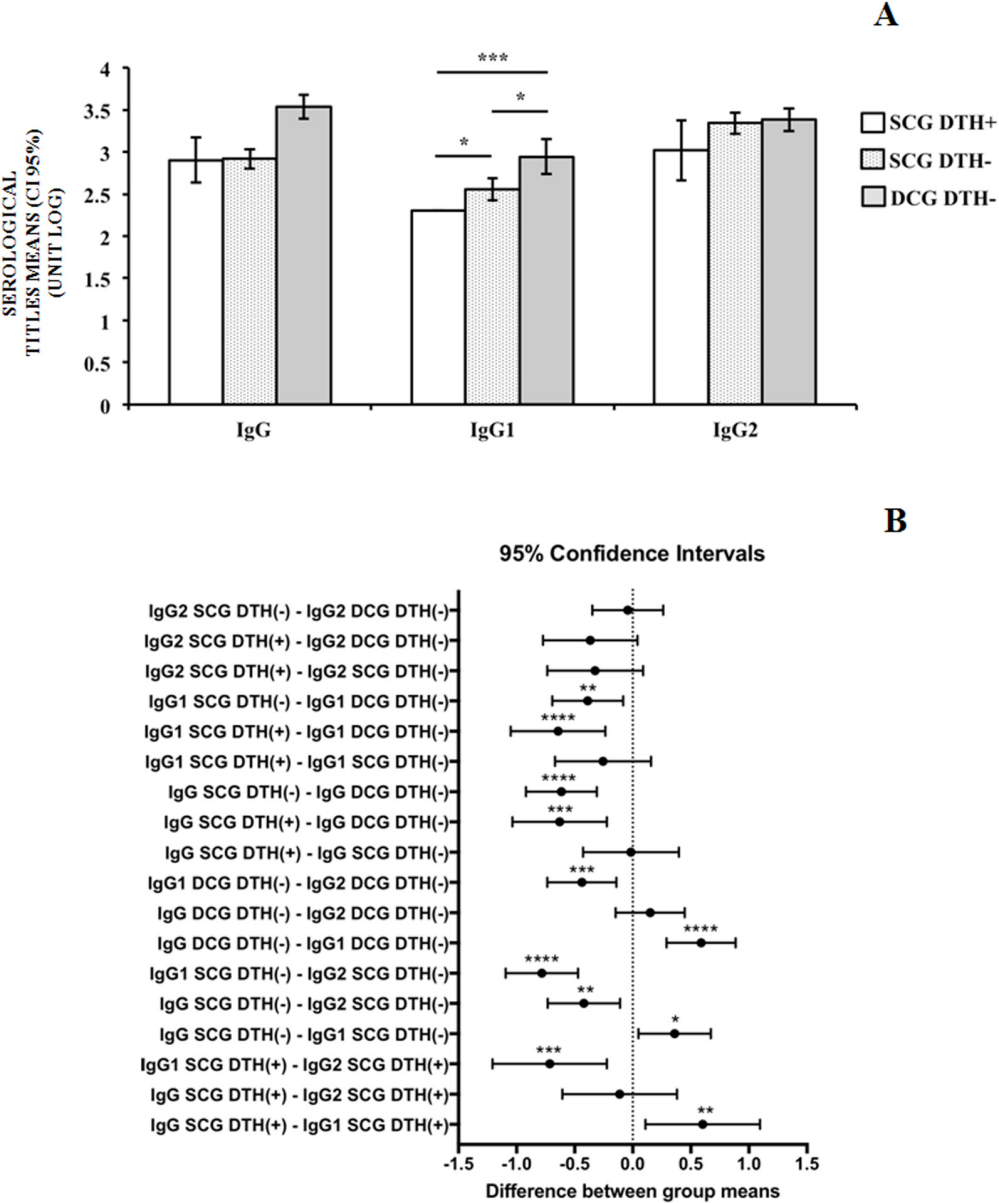
The 95% confidence intervals of the differences between the mean titers of IgG, IgG1, and IgG2 obtained by ELISA, considering the variables of clinical states and DTH results together. The vertical dotted line defines the absence of differences between the means evaluated. ^*^, *p* = 0.0105; ^**^(IgG SCG DTH^(**+**)^** – **IgG1 SCG DTH^(**+**)^), *p* = 0.0054; ^**^(IgG SCG DTH^(**−**)^)** – **IgG2 SCG DTH^(**−**)^), *p* = 0.0012; ^**^(IgG1 SCG DTH^(**−**)^** – **IgG1 DCG DTH^(**−**)^), *p* = 0.0031; ^***^(IgG1 SCG DTH^(**+**)^** – **IgG2 SCG DTH^(**+**)^), *p* = 0.0004; ^***^(IgG1 DCG DTH^(**−**)^** – **IgG2 DCG DTH^(**−**)^), *p* = 0.0003; ^***^(IgG SCG DTH^(**+**)^** – **IgG DCG DTH^(**−**)^), *p* = 0.0001; ^****^, *p* < 0.0001.

Figure 4 demonstrates intra- and intergroup comparisons of IgG, IgG1 and IgG2 serological titer log_10_ values, considering SCG (DTH**^(+)^** and DTH**^(−)^**) animals and DCG (DTH**^(−)^**) animals. On average, intragroup comparisons showed that IgG values were greater than IgG1 values in all groups (SCG DTH**^(+)^**: *p* = 0.0089; SCG DTH**^(−)^**: *p* = 0.0166; DCG DTH**^(−)^**: *p* < 0.0001). The IgG2 values were likewise greater than IgG1 values in all groups (SCG DTH**^(+)^**: *p* = 0.0007; SCG DTH**^(−)^**: *p* < 0.0001; DCG DTH**^(−)^**: *p* = 0.0005). Only within the SCG group (DTH**^(−)^**) were IgG2 values greater than IgG values (SCG DTH**^(−)^**: *p* = 0.0022; SCG DTH**^(+)^**: *p* = 0.9988; DCG DTH**^(−)^**: *p* = 0.8359).

It is also possible to see in Figure 4 that intergroup comparisons showed that, on average, IgG values were greater in the DCG (DTH**^(−)^**) group than in the SCG (DTH**^(+)^**) (*p* = 0.0002) and in the SCG (DTH**^(−)^**) (*p* < 0.0001) groups. Additionally, IgG1 values were greater in the DCG (DTH**^(−)^**) group than in the SCG (DTH**^(+)^**) (*p* = 0.0001) and in the SCG (DTH**^(−)^**) groups (*p* = 0.0053). There were no significant differences between IgG, IgG1 and IgG2 values of SCG (DTH**^(+)^**) and SCG (DTH**^(−)^**) groups (IgG: *p *> 0.9999; IgG1: *p* = 0.626; IgG2: *p* = 0.3019), between IgG2 values of SCG (DTH**^(+)^**) and DCG (DTH**^(−)^**) (*p* = 0.1468) groups, and between IgG2 values of SCG (DTH**^(−)^**) and DCG (DTH**^(−)^**) groups (*p *> 0.9999). However, when the confidence interval of means was also considered, there were verified statistical differences of IgG1 among the analyzed groups (*p* < 0.05) (Figure 4A–B).

**Figure 5:**
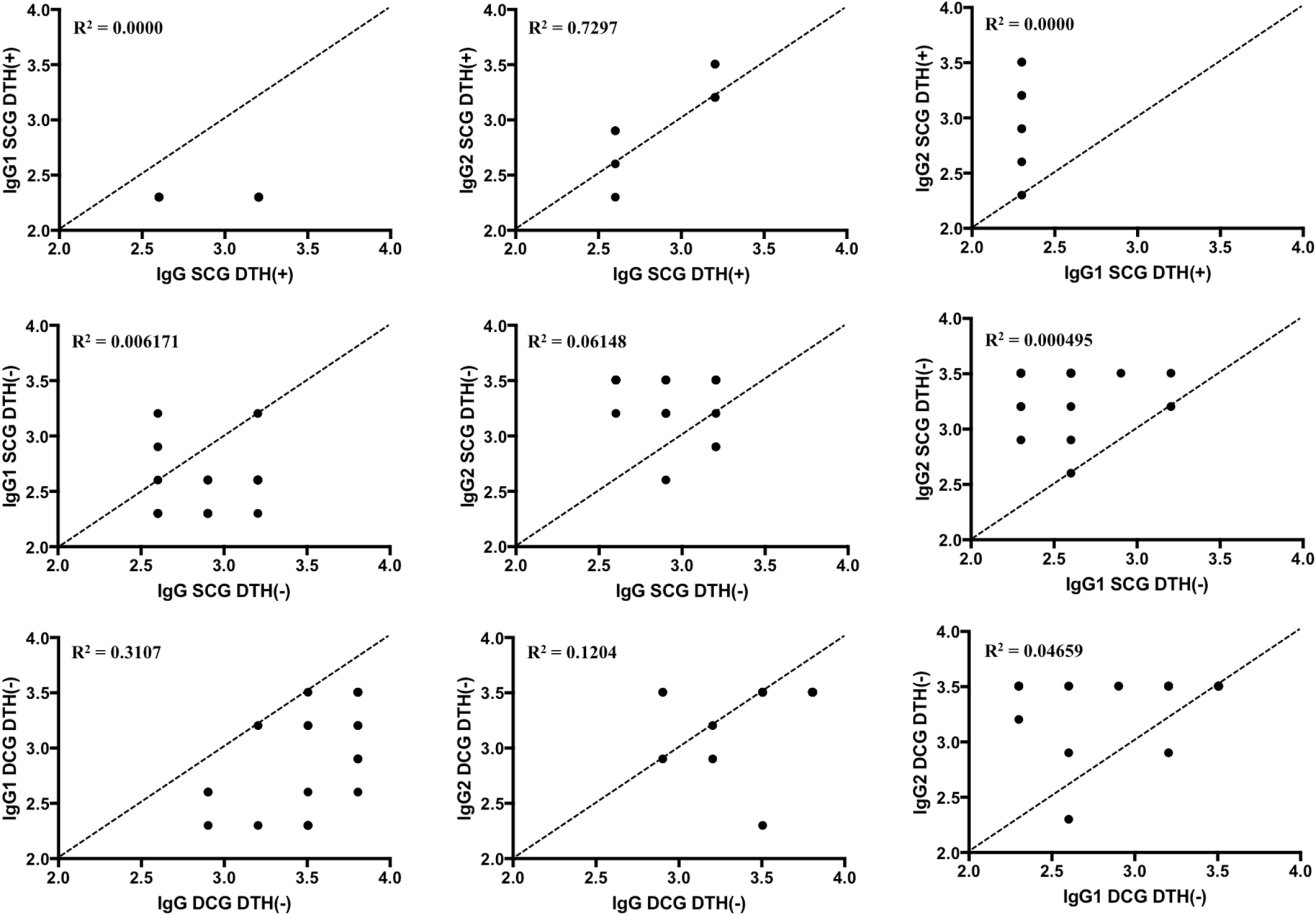
Correlations of log_10_ transformations of IgG, IgG1, and IgG2 sera titer values of the SCG DTH^(**+**)^ group (top), SCG DTH^(**−**)^ group (middle), and DCG DTH^(**−**)^ group (bottom) obtained using ELISA. The diagonal dashed line indicates what would be a perfect positive correlation (*r* = 1) between the variables.

We also evaluated the degree of correlation between serological titers of IgG, IgG1 and IgG2 within the SCG DTH**^(+)^**, SCG DTH**^(−)^** and DCG DTH**^(−)^**groups, and no relevant correlation was found (Figure 5). Nonetheless, the *p* value described in Table 2 suggests that, and not by chance, there is a statistically positive correlation between IgG and IgG2 values (*p* = 0.0069) in the SCG DTH**^(+)^**group. Although the amplitude of the confidence interval of the *r* coefficient (Table 2) indicates that this statistical correlation is not clinically significant, the determination coefficient (R^2^ = 0.7297) indicates that IgG and IgG2 vary in similar manners to the IgG and IgG1 values (R^2^ = 0.0000) in the same group, much as they varied together in the same way as the IgG and IgG2 values in the SCG DTH**^(−)^** (R^2^ = 0.06148) and DCG DTH**^(−)^** (R^2^ = 0.1204) groups. Table 2 also shows that the *p* value suggests, not randomly, that a positive significant statistical correlation was encountered between the IgG and IgG1 (*p* = 0.007) values in the DCG DTH**^(−)^**group. Although the amplitude of the confidence interval of the linear correlation coefficient *r* (see Table 2) indicates that this statistical correlation was not clinically significant, the determination coefficient of that graph (R^2^ = 0.3107) indicates that the IgG and IgG1 values are well correlated, e.g., vary together in a similar fashion, differently from IgG and IgG2 values (R^2^ = 0.1137) in the same group, and also differently from IgG and IgG1 values of the SCG DTH**^(+)^** (R^2^ = 0.0000) and SCG DTH**^(−)^**(R^2^ = 0.006171) groups. Finally, it is also worth noting (Figure 5) that IgG values are generally greater than IgG1 values, and IgG2 values are generally greater than IgG and IgG1 values, corroborating the results seen in Figure 4.

**Table 2:** Correlation analyses between IgG, IgG1, and IgG2 as a function of the variables of clinical state and DTH results.

Statistics	Correlations	SCG DTH(+)	SCG DTH(−)	DCG DTH(−)
*r*	(IgG_,_ IgG1)	(−)	0.07855	0.5574
	(IgG_,_ IgG2)	0.8542	−0.2479	0.347
	(IgG1_,_ IgG2)	(−)	0.02225	0.2158

95% CI	(IgG_,_ IgG1)	(−)	−0.3771 to 0.5036	0.1775 to 0.7927
	(IgG_,_ IgG2)	0.3758 to 0.9731	−0.6222 to 0.2186	−0.08744 to 0.6705
	(IgG1_,_ IgG2)	(−)	−0.4244 to 0.4602	−0.2264 to 0.5843

*p* value	(IgG_,_ IgG1)	(−)	0.742	0.007(^*^)
	(IgG_,_ IgG2)	0.0069(^*^)	0.2919	0.1137
	(IgG1_,_ IgG2)	(−)	0.9258	0.3347

(^*^) Numerically positive linear correlations present, but not significant. (−) Not possible to calculate the *r* values of the confidence interval or the *p* value when the *n* of one of the correlated variables has only one value (Figure 5).

## Discussion

We investigated the likely roles of IgG1 and IgG2 responses as resistance or susceptibility markers of the outcome of CVL in the Brazilian Amazon. Unlike previous work carried out in this region [8,25] or in other areas of Brazil [23], or France [3], and Spain [13], the present work sought to correlate the expression of the IgG1 and IgG2 subclasses not only to the clinical conditions of infected dogs, but also to DTH, which has been recognized as a significant factor of protection against infection [2,5,24,28]. Only the work of Cardoso et al. [6], undertaken in Portugal, associated these IgG subclasses of canine infection with the clinical state and DTH.

Although animal gender has been examined in terms of the clinical state of canine infection with totally negative results [28], we re-examined this variable, testing for any correlation with the expression of the IgG1 and IgG2 subclasses as well as with DTH. Animal gender did not influence infection development with respect to the clinical state or immune responses, whether humoral (IgG1 and IgG2) or T-cell (DTH); similar results were observed during studies of canine infections in Europe [1,20,21].

The evaluation of the IgG, and IgG1 and IgG2 subclasses suggested that the IgG1, but not the IgG2, represents a determinant factor and/or marker of canine susceptibility to disease evolution. When the levels of the IgG1 and IgG2 were compared based on the clinical state of the infection, both SCG and visibly DCG dogs showed significantly greater IgG2 than IgG1 levels. When this comparison considered these clinically different groups of dogs, however, it could be seen that the IgG1 response of the DCG was significantly greater than that of the SCG, while the IgG2 response of dogs in the DCG was similar to that of the SCG. This indicates that the IgG1 response of the SCG was significantly smaller than that of the visibly DCG, as well as the IgG2 response of the dogs in both the SCG and DCG groups. Thus, while the levels of IgG2 were similar among dogs from the two clinical groups, their IgG1 levels differed, being significantly greater in DCG dogs. Additionally, examinations of their IgG levels (and the IgG1 and IgG2 subclasses) indicated that only IgG1 levels were significantly inferior to IgG in both DCG and SCG dogs** – **reinforcing the idea that IgG1 expression is consistently less than IgG and IgG2.

Likewise, comparisons of the levels of IgG and its IgG1 and IgG2 subclasses with the clinical states of these animals showed that there were no significant correlations between the values of these immunoglobulins in SCG and DCG dogs. This indicates that the IgG1 and IgG2 responses represent independent and unequivocal immune-biological events and that IgG1 exercises unappropriate immune response that facilitates not only infection progression, but also its evolution from asymptomatic to symptomatic. An increased level of IgG1 is potentially a marker of this deleterious immune response.

It can therefore be seen that our results diverge from two earlier studies in the same geographic region that suggested that all of the IgG subclasses showed higher levels in DCG than SCG dogs [8,25], but only the IgG1 subclass showed higher levels in the former, while the IgG2 subclass had similar levels in both groups. This also contrasted with reports from São Paulo and Minas Gerais States (southeastern Brazil) [23,26] that suggested that IgG2 is responsible for infection progression.

These discrepancies become more evident when the results are compared with European studies. A number of studies in France, Spain, and Portugal, for example, reported the action of IgG2 with the development of symptomatic infections** – **therefore assuming that IgG2 plays a stronger immunopathogenic role than IgG1 [3,6,13,31]. Deplazes et al. [10], however, suggested the existence of a dichotomy in IgG1 and IgG2 responses in symptomatic and asymptomatic dogs** – **in the sense that IgG2 was associated with asymptomatic and IgG1 with symptomatic infections. Although this interpretation appears similar to that defended here, their results are not in complete agreement with ours. The results demonstrate that IgG2 levels are similar in both DCG and SCG dogs. It is therefore not possible to support the concept of a dichotomy in terms of IgG1 and IgG2 responses to canine infection, but rather a more determinant immunopathogenic action by IgG1 than by IgG2.

This interpretation is strongly supported not only because of the relationships of the IgG1 and IgG2 subclasses with the clinical states of the dogs, but also because of DTH expression in clinically sick (DCG: DTH**^(−)^**) and apparently healthy (SCG: DTH**^(+)^** or DTH**^(−)^**) dogs. Our results demonstrated not only that IgG (and its IgG2 subclass) levels were similar, but that they were significantly greater than IgG1 in all of the groups evaluated when dogs of the same clinical group (sick [IgG1/IgG2 × DCG × DTH**^(−)^**] and apparently healthy [IgG1/IgG2 ×* *SCG ×* *DTH**^(+)^** or DTH**^(−)^**]) were compared. When this comparison was made between dogs of two clinically different groups, however, IgG1 levels of DCG (DTH**^(−)^**) dogs were significantly greater than the IgG1 levels of SCG (DTH**^(−)^** or DTH**^(+)^**) animals.

Additionally, although the IgG1 levels of SCG (DTH**^(−)^**) dogs were not statistically greater than those of SCG (DTH**^(+)^**) animals, when the confidence intervals of the differences between their IgG1 means were considered (Figure 4, bottom), the apparent absence of statistical differences could be explained by the fact that this analysis did not consider the variability of IgG1 levels in the samples, but only the differences between mean IgG1 levels. As the mean IgG1 values of the eight SCG (DTH**^(+)^**) dogs were exactly equal, the mean IgG1 value of this group generated a mean, without sample variability. As such, the solution for this inconsistency was to analyze the difference between the 95% confidence intervals of the means, an analysis that centers on the variability of the IgG1 values in the samples of the SCG (DTH**^(−)^**) and SCG (DTH**^(+)^**) groups, allowing us to evaluate the true differences between their IgG1 levels (Figure 4, top). It can then be seen that there was a strong tendency for the IgG1 levels of SCG (DTH**^(−)^**) dogs to be greater than those of SCG (DTH**^(+)^**) animals, suggesting that the IgG1 levels of SCG (DTH**^(−)^**) dogs were greater than those of SCG (DTH**^(+)^**) animals. IgG1 levels also progressively increased from SCG (DTH**^(+)^**) dogs, to SCG (DTH**^(−)^**), and finally to DCG (DTH**^(−)^**) animals. This showed a close relationship between IgG1 levels and DTH responses. It thus appears that in SCG dogs expressing positive DTH, IgG1 expression is controlled by the T-cell response (DTH**^(+)^**), while in dogs showing a negative T-cell response [i.e., SCG (DTH**^(−)^**) and DCG dogs (DTH**^(−)^**)], IgG1 expression would be less controlled (more evident).

Confirming these observations is the fact that there were no strong correlations between IgG levels (and their IgG1 and IgG2 subclasses) and the clinical states of the dogs and their DTH expressions in either of the groups analyzed. This was the case despite the fact that these SCG DTH**^(+)^** and DCG DTH**^(−)^** groups demonstrated statistically positive correlations between their IgG and IgG2, and IgG and IgG1 values, indicating that the IgG1 levels of SCG DTH**^(+)^**/DTH**^(−)^** dogs were lower than those of DCG DTH**^(−)^**dogs (as well as their IgG and IgG2 levels).

As noted earlier, the results of Cardoso et al. [7] were similar to those observed in the present work reinforcing our proposal that the DTH**^(+)^** of the infected dogs must be controlling the expression of the IgG1 and IgG2 subclasses, in spite of the fact that IgG2 expression in sick dogs (DCG: DTH**^(−)^**) and in apparently healthy animals (SCG: DTH**^(+)^**/DTH**^(−)^**) are similar to (although higher than) IgG1 expression. Santos et al. [11] also demonstrated for Brazil that dogs with positive splenic cultures and DTH**^(−)^** had higher levels of IgG and IgG2 than dogs with negative splenic cultures and DTH**^(+)^**.

## Funding

This research was supported by the Evandro Chagas Institute (Ministry of Health, Brazil) and Nucleus of Tropical Medicine (Federal University of Pará State, Brazil).

## Conflict of interest

The authors declare that they have no conflicts of interest in relation to this article.
